# Supplementary material for: Network analysis of rare single-nucleotide polymorphisms suggests a central role for type 17 immune responses in trauma-induced, genotypically associated hypo-inflammation and critical illness
Source: Front Syst Biol. 2026 Oct 28;6:1720950. doi: 10.3389/fsysb.2026.1720950 (PMC13751389; doi:10.3389/fsysb.2026.1720950)
Supplement: Supplementary file 2 [file DataSheet2.pdf]

SUPPLEMENTARY TABLES

Supplementary Table 1. Evenly distributed control SNPs with 3-4 statistically distinct post-traumatic outcomes between AA and BB genotypes ($p < 0.05$).

SNP	ICU Days	Total Hospital Days	Vent Days	Marshall Mods	Gene Found with FLAME
rs992708	✓	✓	✓	✓	SNX7
rs10865443	✓	✓	✓	✓	CTNNA2
exm2263860	✓	✓	✓	✓	---
rs13078747	✓	✓	✓	✓	CACNA1D
rs704716	✓	✓	✓	✓	B3GALNT2
rs4771918	✓		✓	✓	CLDN10, CLDN10-AS1
rs6932393	✓	✓		✓	CDKAL1
exm-rs89107		✓	✓	✓	---
rs12986520		✓	✓	✓	LINC00276
rs1671471	✓	✓		✓	---
rs896973	✓		✓	✓	TPCN2, ENSG00000287725
rs4685385	✓	✓		✓	---
rs7701186	✓	✓		✓	---
rs7967371	✓	✓		✓	---
rs719680	✓		✓	✓	---
rs12127469		✓	✓	✓	ZRANB2-DT

Supplementary Table 2. Rare control SNPs with 3-4 statistically distinct post-traumatic outcomes between AA and BB genotypes ($p < 0.05$).

SNP	ICU Days	Total Hospital Days	Vent Days	Marshall Mods	Gene Found with FLAME
rs1409117	✓	✓	✓	✓	IQSEC2
rs2388252	✓	✓	✓	✓	---
rs12450000	✓	✓		✓	---
rs4144013		✓	✓	✓	PPRG1
rs6609469	✓	✓		✓	---
rs9558839	✓	✓		✓	---
rs7609779	✓		✓	✓	ABI3BP
rs767478	✓	✓		✓	---
rs841852	✓	✓		✓	SLC2A1
rs7514892		✓	✓	✓	RGS21

Supplementary Table 3. FLAME results of evenly distributed control SNPs with *gProfiler*.

Source	Term_ID	Function	P-value	Enrichment Score %	Positive Hits
GO:MF	GO:0022832	voltage-gated channel activity	3.28E-03	1.61	CACNA1D, ENSG00000287725, TPCN2
GO:MF	GO:0005245	voltage-gated calcium channel activity	5.22E-03	3.77	CACNA1D, TPCN2
GO:MF	GO:0022836	gated channel activity	5.22E-03	0.95	CACNA1D, ENSG00000287725, TPCN2
GO:MF	GO:0086059	voltage-gated calcium channel activity involved SA node cell action potential	5.78E-03	50	CACNA1D
GO:MF	GO:0022803	passive transmembrane transporter activity	5.78E-03	0.59	CACNA1D, ENSG00000287725, TPCN2
GO:MF	GO:0005216	monoatomic ion channel activity	5.78E-03	0.68	CACNA1D, ENSG00000287725, TPCN2
GO:MF	GO:0015267	channel activity	5.78E-03	0.59	CACNA1D, ENSG00000287725, TPCN2
GO:MF	GO:0035598	N6-threonylcarbomyladenosine methylthiotransferase activity	5.78E-03	100	CDKAL1
GO:MF	GO:0050497	alkylthioltransferase activity	5.78E-03	50	CDKAL1
GO:MF	GO:0035596	methylthiotransferase activity	5.78E-03	50	CDKAL1
GO:MF	GO:0061712	tRNA (N(6)-L-threonylcarbamoyladenosine(37)-C(2))-methylthiotransferase	5.78E-03	100	CDKAL1
GO:MF	GO:0005262	calcium channel activity	7.04E-03	1.57	CACNA1D, TPCN2
GO:MF	GO:0097682	intracellular phosphatidylinositol-3,5-bisphosphate-sensitive monatomic cation channel activity	7.21E-03	33.33	TPCN2
GO:MF	GO:0015085	calcium ion transmembrane transporter activity	7.85E-03	1.38	CACNA1D, TPCN2
GO:MF	GO:0022843	voltage-gated monoatomic cation channel activity	8.47E-03	1.28	CACNA1D, TPCN2
GO:MF	GO:0086007	voltage-gated calcium channel activity involved in cardiac muscle cell action potential	9.19E-03	20	CACNA1D
GO:MF	GO:0072345	NAADP-sensitive calcium-release channel activity	9.19E-03	20	TPCN2
GO:MF	GO:0015075	monoatomic ion transmembrane transporter activity	9.42E-03	0.41	CACNA1D, ENSG00000287725, TPCN2
GO:MF	GO:0005244	voltage-gated monoatomic ion channel activity	9.42E-03	1.09	CACNA1D, TPCN2

GO:MF	GO:0035091	phosphatidylinositol binding	1.96E-02	0.72	SNX7, TPCN2
GO:MF	GO:0022857	transmembrane transporter activity	2.48E-02	0.27	CACNA1D, ENSG00000287725, TPCN2
GO:MF	GO:0005261	monoatomic cation channel activity	2.47E-02	0.6	CACNA1D, TPCN2
GO:MF	GO:0030506	ankyrin binding	2.47E-02	5.26	CACNA1D
GO:MF	GO:0015278	calcium-release channel activity	2.47E-02	5.56	TPCN2
GO:MF	GO:0015280	ligand-gated sodium channel activity	2.47E-02	5.88	TPCN2
GO:MF	GO:0005215	transporter activity	2.90E-02	0.24	CACNA1D, ENSG00000287725, TPCN2
GO:MF	GO:0051393	alpha-actinin binding	2.90E-02	3.57	CACNA1D
GO:MF	GO:0008331	high voltage-gated calcium channel activity	2.87E-02	4.17	CACNA1D
GO:MF	GO:0080025	phosphatidylinositol-3,5-bisphosphate binding	2.90E-02	3.7	TPCN2
GO:MF	GO:0099604	ligand-gated calcium channel activity	2.90E-02	3.7	TPCN2
GO:MF	GO:0005217	intracellular ligand-gated monoatomic ion channel activity	3.01E-02	3.33	TPCN2
GO:MF	GO:0046873	metal ion transmembrane transporter activity	3.06E-02	0.45	CACNA1D, TPCN2
GO:MF	GO:0008376	acetylgalactosaminyltransferase activity	3.20E-02	2.94	B3GALNT2
GO:MF	GO:0042805	actinin binding	3.28E-02	2.7	CACNA1D
GO:MF	GO:0005543	phospholipid binding	3.28E-02	0.41	SNX7, TPCN2
GO:MF	GO:0005272	sodium channel activity	3.71E-02	2.33	TPCN2
GO:MF	GO:0051539	4 iron, 4 sulfur cluster binding	3.77E-02	2.22	CDKAL1
GO:MF	GO:0022890	inorganic cation transmembrane transporter activity	4.11E-02	0.35	CACNA1D, TPCN2
KEGG	map05412	Arrhythmogenic right ventricular cardiomyopathy	4.27E-02	2.38	CACNA1D, CTNNA2
KEGG	map04670	Leukocyte transendothelial migration	4.27E-02	1.75	CLDN10, CTNNA2
GO:MF	GO:0008324	monoatomic cation transmembrane transporter activity	4.54E-02	0.33	CACNA1D, TPCN2

Supplementary Table 4. FLAME results of evenly distributed control SNPs with *enrichR*.

Source	Term_ID	Function	P-value	Enrichment Score %	Positive Hits
GO:MF	GO:0005245	voltage-gated calcium channel activity	2.14E-03	5.41	CACNA1D, TPCN2
GO:MF	GO:0005262	calcium channel activity	4.92E-03	2.38	CACNA1D, TPCN2

GO:MF	GO:0022843	voltage-gated cation channel activity	4.92E-03	2.06	CACNA1D, TPCN2
GO:MF	GO:0086007	voltage-gated calcium channel activity involved in cardiac muscle cell action potential	8.09E-03	20	CACNA1D
GO:MF	GO:0072345	NAADP-sensitive calcium-release channel activity	8.09E-03	20	TPCN2
GO:MF	GO:0008331	high voltage-gated calcium channel activity	1.35E-02	10	CACNA1D
KEGG	map05412	Arrhythmogenic right ventricular cardiomyopathy (ARVC)	1.72E-02	2.7	CACNA1D, CTNNA2
GO:MF	GO:0030506	ankyrin binding	1.88E-02	5.26	CACNA1D
GO:MF	GO:0042805	actinin binding	1.88E-02	4.76	CACNA1D
GO:MF	GO:0015278	calcium-release channel activity	1.88E-02	5.88	TPCN2
GO:MF	GO:0051393	alpha-actinin binding	1.94E-02	4.17	CACNA1D
GO:MF	GO:0008376	acetylgalactosaminyltransferase activity	1.98E-02	3.7	B3GALNT2
KEGG	map04670	Leukocyte transendothelial migration	2.01E-02	1.69	CLDN10, CTNNA2
KEGG	map04530	Tight junction	2.01E-02	1.44	CLDN10, CTNNA2
GO:BP	GO:0086015	SA node cell action potential	3.22E-02	14.29	CACNA1D
GO:BP	GO:0086046	membrane depolarization during SA node cell action potential	3.22E-02	20	CACNA1D
GO:BP	GO:0045762	positive regulation of adenylate cyclase activity	3.22E-02	14.29	CACNA1D
GO:BP	GO:0060372	regulation of atrial cardiac muscle cell membrane repolarization	3.22E-02	14.29	CACNA1D
GO:BP	GO:0060134	prepulse inhibition	3.22E-02	16.67	CTNNA2
GO:BP	GO:0034316	negative regulation of Arp2/3 complex-mediated actin nucleation	3.22E-02	16.67	CTNNA2
GO:BP	GO:0051823	regulation of synapse structural plasticity	3.22E-02	20	CTNNA2
GO:BP	GO:0019065	receptor-mediated endocytosis of virus by host cell	3.22E-02	14.29	TPCN2
GO:BP	GO:0051126	negative regulation of actin nucleation	3.28E-02	12.5	CTNNA2
GO:MF	GO:0015085	calcium ion transmembrane transporter activity	3.67E-02	1.82	CACNA1D
GO:BP	GO:0086012	membrane depolarization during cardiac muscle cell action potential	3.76E-02	5.88	CACNA1D
GO:BP	GO:0086002	cardiac muscle cell action potential involved in contraction	3.76E-02	3.23	CACNA1D
GO:BP	GO:0045761	regulation of adenylate cyclase activity	3.76E-02	4.55	CACNA1D
GO:BP	GO:0070509	calcium ion import	3.76E-02	3.57	CACNA1D

GO:BP	GO:0051349	positive regulation of lyase activity	3.76E-02	6.67	CACNA1D
GO:BP	GO:0086001	cardiac muscle cell action potential	3.76E-02	3.33	CACNA1D
GO:BP	GO:0086003	cardiac muscle cell contraction	3.76E-02	5.26	CACNA1D
GO:BP	GO:0086010	membrane depolarization during action potential	3.76E-02	3.85	CACNA1D
GO:BP	GO:0086091	regulation of heart rate by cardiac conduction	3.76E-02	2.56	CACNA1D
GO:BP	GO:1904062	regulation of cation transmembrane transport	3.76E-02	3.33	CACNA1D
GO:BP	GO:0031281	positive regulation of cyclase activity	3.76E-02	7.14	CACNA1D
GO:BP	GO:0043266	regulation of potassium ion transport	3.76E-02	3.23	CACNA1D
GO:BP	GO:0051928	positive regulation of calcium ion transport	3.76E-02	2.7	CACNA1D
GO:BP	GO:0099623	regulation of cardiac muscle cell membrane repolarization	3.76E-02	4.55	CACNA1D
GO:BP	GO:1901016	regulation of potassium ion transmembrane transporter activity	3.76E-02	3.85	CACNA1D
GO:BP	GO:0032412	regulation of ion transmembrane transporter activity	3.76E-02	2.56	CACNA1D
GO:BP	GO:0016338	calcium-independent cell-cell adhesion via plasma membrane cell-adhesion molecules	3.76E-02	5	CLDN10
GO:BP	GO:0051049	regulation of transport	3.76E-02	2.7	CLDN10
GO:BP	GO:0034315	regulation of Arp2/3 complex-mediated actin nucleation	3.76E-02	5.88	CTNNA2
GO:BP	GO:0048813	dendrite morphogenesis	3.76E-02	2.78	CTNNA2
GO:BP	GO:0050807	regulation of synapse organization	3.76E-02	3.85	CTNNA2
GO:BP	GO:0051147	regulation of muscle cell differentiation	3.76E-02	2.86	CTNNA2
GO:BP	GO:0120035	regulation of plasma membrane bounded cell projection organization	3.76E-02	3.57	CTNNA2
GO:BP	GO:2001222	regulation of neuron migration	3.76E-02	3.57	CTNNA2
GO:BP	GO:0048854	brain morphogenesis	3.76E-02	8.33	CTNNA2
GO:BP	GO:0051149	positive regulation of muscle cell differentiation	3.76E-02	3.7	CTNNA2
GO:BP	GO:0006939	smooth muscle contraction	3.76E-02	7.14	TPCN2
GO:BP	GO:0007040	lysosome organization	3.76E-02	2.63	TPCN2
GO:BP	GO:0080171	lytic vacuole organization	3.76E-02	3.03	TPCN2
GO:BP	GO:0043270	positive regulation of ion transport	4.68E-02	2	CACNA1D
GO:BP	GO:1901379	regulation of potassium ion	4.81E-02	1.85	CACNA1D

		transmembrane transport			
GO:BP	GO:0051924	regulation of calcium ion transport	4.81E-02	1.72	CACNA1D
GO:BP	GO:0009451	RNA modification	4.81E-02	1.72	CDKAL1
GO:BP	GO:0070830	bicellular tight junction assembly	4.81E-02	1.89	CLDN10
GO:BP	GO:0120192	tight junction assembly	4.81E-02	1.79	CLDN10
GO:BP	GO:0006400	tRNA modification	4.93E-02	1.54	CDKAL1
GO:BP	GO:0008033	tRNA processing	4.93E-02	1.56	CDKAL1
GO:BP	GO:0043297	apical junction assembly	4.86E-02	1.67	CLDN10
GO:BP	GO:0031329	regulation of cellular catabolic process	4.93E-02	1.54	TPCN2

Supplementary Table 5. FLAME results of rare control SNPs with *gProfiler*.

Source	Term_ID	Function	P-value	Enrichment Score %	Positive Hits
GO:CC	GO:1990350	glucose transporter complex	2.09E-02	100	SLC2A1
GO:BP	GO:1905204	negative regulation of connective tissue replacement	2.75E-02	50	ABI3BP
GO:BP	GO:1905203	regulation of connective tissue replacement	2.75E-02	16.67	ABI3BP
GO:BP	GO:1904597	negative regulation of connective tissue replacement involved in inflammatory response wound healing	2.75E-02	50	ABI3BP
GO:BP	GO:1904596	regulation of connective tissue replacement involved in inflammatory response wound healing	2.75E-02	50	ABI3BP
GO:BP	GO:0002248	connective tissue replacement involved in inflammatory response wound healing	2.75E-02	16.67	ABI3BP
GO:BP	GO:1900454	positive regulation of long-term synaptic depression	2.75E-02	14.29	IQSEC2
GO:BP	GO:0050916	sensory perception of sweet taste	2.75E-02	14.29	RGS21
GO:BP	GO:0050917	sensory perception of umami taste	2.75E-02	14.29	RGS21
GO:BP	GO:0140271	hexose import across plasma membrane	2.75E-02	16.67	SLC2A1
GO:BP	GO:1904016	response to Thyroglobulin triiodothyronine	2.75E-02	20	SLC2A1
GO:BP	GO:0098708	glucose import across plasma membrane	2.75E-02	20	SLC2A1
GO:BP	GO:0098704	carbohydrate import across plasma membrane	2.75E-02	16.67	SLC2A1
GO:BP	GO:0002246	wound healing involved in inflammatory response	2.90E-02	12.5	ABI3BP

GO:BP	GO:0097709	connective tissue replacement	2.98E-02	8.33	ABI3BP
GO:BP	GO:0098696	regulation of neurotransmitter receptor localization to postsynaptic specialization membrane	2.98E-02	8.33	IQSEC2
GO:BP	GO:1901334	lactone metabolic process	2.98E-02	9.09	SLC2A1
GO:BP	GO:0070837	dehydroascorbic acid transport	2.98E-02	11.11	SLC2A1
GO:BP	GO:0015911	long-chain fatty acid import across plasma membrane	2.98E-02	10	SLC2A1
GO:BP	GO:0019852	L-ascorbic acid metabolic process	2.98E-02	9.09	SLC2A1
GO:BP	GO:1905209	positive regulation of cardiocyte differentiation	3.06E-02	7.69	ABI3BP
GO:BP	GO:1900452	regulation of long-term synaptic depression	3.37E-02	6.67	IQSEC2
GO:BP	GO:0034104	negative regulation of tissue remodeling	3.61E-02	4.35	ABI3BP
GO:BP	GO:1902473	regulation of protein localization to synapse	3.61E-02	4.35	IQSEC2
GO:BP	GO:1902683	regulation of receptor localization to synapse	3.61E-02	4.55	IQSEC2
GO:BP	GO:0032011	ARF protein signal transduction	3.61E-02	4.55	IQSEC2
GO:BP	GO:0032012	regulation of ARF protein signal transduction	3.61E-02	4.55	IQSEC2
GO:BP	GO:0044539	long-chain fatty acid import into cell	3.61E-02	5.88	SLC2A1
GO:BP	GO:0140354	lipid import into cell	3.61E-02	5	SLC2A1
GO:BP	GO:0071474	cellular hyperosmotic response	3.61E-02	5	SLC2A1
GO:BP	GO:1902001	fatty acid transmembrane transport	3.61E-02	5	SLC2A1
GO:BP	GO:1905207	regulation of cardiocyte differentiation	3.77E-02	4	ABI3BP
GO:BP	GO:0090594	inflammatory response to wounding	3.77E-02	3.85	ABI3BP
GO:BP	GO:0099633	protein localization to postsynaptic specialization membrane	3.77E-02	3.57	IQSEC2
GO:BP	GO:0099645	neurotransmitter receptor localization to postsynaptic specialization membrane	3.77E-02	3.57	IQSEC2
GO:BP	GO:0006972	hyperosmotic response	3.77E-02	3.7	SLC2A1
GO:BP	GO:0060292	long-term synaptic depression	4.31E-02	3.03	IQSEC2
GO:BP	GO:0051968	positive regulation of synaptic transmission, glutamatergic	4.58E-02	2.78	IQSEC2
KEGG	map04066	HIF-1 signaling pathway	4.72E-02	0.92	SLC2A1
KEGG	map04931	Insulin resistance	4.72E-02	0.93	SLC2A1
KEGG	map04911	Insulin secretion	4.72E-02	1.18	SLC2A1

KEGG	map04920	Adipocytokine signaling pathway	4.72E-02	1.43	SLC2A1
KEGG	map04919	Thyroid hormone signaling pathway	4.72E-02	0.83	SLC2A1
KEGG	map04976	Bile secretion	4.72E-02	1.12	SLC2A1
KEGG	map04922	Glucagon signaling pathway	4.72E-02	0.93	SLC2A1
KEGG	map05230	Central carbon metabolism in cancer	4.72E-02	1.43	SLC2A1
KEGG	map05211	Renal cell carcinoma	4.72E-02	1.47	SLC2A1

Supplementary Table 6. FLAME results of rare control SNPs with *enrichR*

Source	Term_ID	Function	P-value	Enrichment Score %	Positive Hits
GO:MF	GO:0015149	hexose transmembrane transporter activity	1.05E-02	6.67	SLC2A1
GO:MF	GO:0005355	glucose transmembrane transporter activity	1.05E-02	4.76	SLC2A1
GO:BP	GO:0046351	disaccharide biosynthetic process	1.25E-02	20	SLC2A1
GO:BP	GO:0098708	glucose import across plasma membrane	1.25E-02	20	SLC2A1
GO:BP	GO:0140271	hexose import across plasma membrane	1.25E-02	16.67	SLC2A1
GO:BP	GO:0019852	L-ascorbic acid metabolic process	1.25E-02	11.11	SLC2A1
GO:BP	GO:0044539	long-chain fatty acid import into cell	1.25E-02	10	SLC2A1
GO:BP	GO:0046323	glucose import	1.35E-02	7.69	SLC2A1
GO:BP	GO:0010827	regulation of glucose transmembrane transport	1.39E-02	5.26	SLC2A1
GO:BP	GO:1902001	fatty acid transmembrane transport	1.39E-02	5.26	SLC2A1
GO:BP	GO:1904659	glucose transmembrane transport	1.39E-02	5	SLC2A1
GO:BP	GO:0008645	hexose transmembrane transport	1.43E-02	4.35	SLC2A1
GO:BP	GO:0099177	regulation of trans-synaptic signaling	1.82E-02	2.86	IQSEC2
GO:BP	GO:0034762	regulation of transmembrane transport	1.82E-02	2.94	SLC2A1
GO:BP	GO:0019752	carboxylic acid metabolic process	1.92E-02	2.5	SLC2A1
GO:BP	GO:0090276	regulation of peptide hormone secretion	3.13E-02	1.35	SLC2A1
GO:BP	GO:0006767	water-soluble vitamin metabolic process	3.13E-02	1.32	SLC2A1
GO:BP	GO:0010232	vascular transport	3.13E-02	1.19	SLC2A1
GO:BP	GO:0150104	transport across blood-brain barrier	3.13E-02	1.16	SLC2A1

GO:BP	GO:0050804	modulation of chemical synaptic transmission	3.46E-02	0.92	IQSEC2
GO:BP	GO:0050796	regulation of insulin secretion	3.46E-02	0.96	SLC2A1
GO:BP	GO:0043933	protein-containing complex subunit organization	3.46E-02	0.89	SLC2A1
GO:BP	GO:0050708	regulation of protein secretion	3.67E-02	0.8	SLC2A1
KEGG	map05230	Central carbon metabolism in cancer	3.89E-02	1.49	SLC2A1
KEGG	map04976	Bile secretion	3.89E-02	1.41	SLC2A1
KEGG	map04922	Glucagon signaling pathway	3.89E-02	0.99	SLC2A1
KEGG	map05211	Renal cell carcinoma	3.89E-02	1.52	SLC2A1
KEGG	map04920	Adipocytokine signaling pathway	3.89E-02	1.43	SLC2A1
KEGG	map04931	Insulin resistance	3.89E-02	0.92	SLC2A1
KEGG	map04911	Insulin secretion	3.89E-02	1.18	SLC2A1
KEGG	map04066	HIF-1 signaling pathway	3.89E-02	0.97	SLC2A1
KEGG	map04919	Thyroid hormone signaling pathway	3.89E-02	0.85	SLC2A1
GO:CC	GO:0030864	cortical actin cytoskeleton	4.33E-02	2.38	SLC2A1
GO:CC	GO:0042383	sarcolemma	4.33E-02	1.92	SLC2A1
GO:CC	GO:0030863	cortical cytoskeleton	4.33E-02	1.72	SLC2A1
GO:MF	GO:0005085	guanyl-nucleotide exchange factor activity	4.89E-02	0.67	IQSEC2
